# Neural dynamics and acoustic adaptations during the Lombard effect: evidence from EEG and dynamic causal modeling

**DOI:** 10.1038/s41598-026-49995-x

**Published:** 2026-04-24

**Authors:** Lucía Z-Rivera, Christian Castro, Jhosmary Cuadros, Juan P. Cortés, Alejandro Weinstein, Víctor M. Espinoza, Pavel Prado, Matías Zañartu

**Affiliations:** 1 Advanced Center for Electrical and Electronic Engineering, Universidad Técnica Federico Santa María, Valparaíso, Chile; 2 Neuroscience and Cognitive Science Program, University of Maryland, College Park, MD USA; 3 Department of Human Development and Quantitative Methodology, University of Maryland, College Park, MD USA; 4 Exercise and Rehabilitation Sciences Institute, School of Speech Therapy, Faculty of Rehabilitation Sciences, Universidad Andres Bello, Santiago, 7591538 Chile; 5 Ph.D. Program in Health Science and Engineering Universidad de Valparaíso, Valparaíso, Chile; 6 Latin American Brain Health Institute (BrainLat), Universidad Adolfo Ibañez, Santiago, Chile; 7 Grupo de Bioingeniería, Decanato de Investigación, Universidad Nacional Experimental del Táchira, San Cristóbal 5001, Venezuela; 8 Department of Electronic Engineering, Universidad Técnica Federico Santa María, Valparaíso, Chile; 9 Department of Sound, Universidad de Chile, Santiago, Chile; 10 Escuela de Fonoaudiología, Facultad de Ciencias de la Rehabilitación y Calidad de Vida, Universidad San Sebastián, Santiago, Chile

**Keywords:** Neuroscience, Physiology

## Abstract

The Lombard Effect (LE) is a vocal adaptation in which speakers involuntarily increase their vocal effort to preserve intelligibility in high-noise environments. This study explores acoustic and neurophysiological mechanisms that support LE in individuals with typical voices. Twenty-one participants produced 80 syllables under three conditions: Baseline (quiet), Lombard (noise at 80 dB SPL), and Recovery (quiet after five minutes of rest). Acoustic signals and electroencephalography (EEG) data were recorded synchronously, focusing on sound pressure level (SPL), H1–H2 (the difference in amplitude between the first and second harmonics), Cepstral Peak Prominence (CPP), Event-Related Potentials (ERPs) time-locked to the onset of self-produced vocalizations, and effective connectivity through Dynamic Causal Modeling (DCM). Results showed a significant increase in SPL during the Lombard condition compared to Baseline and Recovery. In this condition, H1–H2 values decreased and CPP increased, with no differences between Baseline and Recovery across the three acoustic measures. ERP analysis revealed a higher N1-P2 amplitude in the Lombard condition, associated with increased activations in frontal, limbic, and temporal brain regions. Bayesian model selection within the DCM framework indicated that the best-fitting model explaining the ERP data was a forward network from the primary auditory cortex (A1) to the temporal pole (TPO), inferior frontal gyrus (IFG), and parahippocampal gyrus (PHG), with modulatory connections highlighting feedback mechanisms. Within this network, the IFG and PHG seem to play a central role in error detection and feedback modulation, while the TPO supports auditory processing, together supporting the neural adjustments that sustain intelligible speech in noise. These results provide new insights into the cortical network underlying LE, emphasizing the adaptive mechanisms in speech production under noisy conditions.

## Introduction

In numerous everyday circumstances, oral communication occurs in acoustic environments where ambient noise exceeds conversational levels. In such situations, speakers increase their vocal effort involuntarily to preserve the clarity, intelligibility, and comprehension of speech^1,2^. This behavioral adaptation is defined as the Lombard Effect (LE).

The LE incorporates various acoustic and phonemic changes in voice production, including increases in sound pressure level (SPL), fundamental frequency, and modifications in the duration of vowels and syllables^3,4^. Moreover, LE involves changes in the spectral distribution of acoustic energy, with increases in energy within the second and third formant regions^5^. The LE typically emerges when the intensity of the background noise surpasses 43 dB SPL^6^. As noise levels increase beyond 55 dB SPL, voice SPL rises by approximately 0.38 dB for every additional dB of background noise^7,8^. Moreover, LE is more pronounced when background noise has a similar spectral composition to speech, compared to noise without speech-like frequencies^8,9^. Essentially, LE acts as an adaptive communication mechanism influenced by the linguistic context of the message^10^, the communicative intent and interaction between speakers and their audience^9^, and the spectral composition of the masking noise^8,11^.

Neuroanatomic evidence from animal models suggests that the LE is primarily elicited by subcortical structures and modulated by cortical processes^12^. In humans, subcortical regions such as the thalamus and right pallidum contribute to motor control during speech, particularly under noisy conditions, compared to speaking in silence^11^. The cerebellum is also recruited, together with cortical areas including the superior temporal gyrus (STG), prefrontal cortex, left insula, and posterior parietal cortex. Greater bilateral activation in STG has been observed when the masker has the spectrotemporal complexity of speech and lexical information, suggesting the need for linguistic processing resources^13^. In addition, the prefrontal and posterior parietal regions show increased activity, reflecting the attentional demands required to suppress background noise and enhance speech intelligibility^14^.

These cortical and subcortical regions work together to manage the complex interplay of predictive and feedback mechanisms during speech production. Previous evidence suggests that the supplementary motor area sends signals to motor control regions to produce target speech while simultaneously generating predictive inhibitory signals to the auditory and somatosensory cortices^15^. When auditory feedback matches predictions, it is ignored. However, interference can create a mismatch between predictive and actual feedback, triggering an error signal that prompts real-time corrective adjustments in speech muscles^16,17^. Masking noise represents a form of auditory interference that can disrupt the precision of feedback-based control^12,15,18^. This comparison and correction mechanism has been implemented in computational frameworks, most prominently in the “Directions into Velocities of Articulators model” (DIVA), which describes how auditory areas detect prediction–feedback mismatches and transmit error signals to frontal motor regions to implement immediate corrections^19,20^.

Moreover, if the same type of error persists across multiple speech repetitions, the original speech motor plans might be updated, resulting in long-term adaptation^16,17,21^. The involuntary increase in vocal effort observed in LE may thus act as both an immediate corrective response to the error signal and, over time, contribute to adjustments in motor plans as part of this adaptive process^22^.

Research on the LE has mainly relied on acoustic–behavioral analyses^3,4,6,8–10,23,24^ and indirect neuroimaging methods^11,25,26^. However, these approaches have limitations, particularly because techniques such as functional magnetic resonance imaging (fMRI) capture hemodynamic rather than direct neural activity. Electroencephalography (EEG) helps address this limitation by providing a direct measure of the brain’s electrical activity, allowing for a more precise characterization of the neural dynamics associated with the LE. EEG also complements the spatial information obtained from fMRI^27^. Notably, no EEG-based studies have yet investigated the LE, and this article presents the first attempt to do so using this method.

EEG provides an effective tool for investigating the dynamics of vocal production under altered auditory feedback conditions^28–33^. For example, Behroozmand et al. employed EEG to study both reflexive and adaptive mechanisms in response to pitch-shifted auditory feedback perturbations during vocal production. Analyzing Event-Related Potentials (ERPs) in their reflexive paradigm, they found aligned vocal responses and reduced N1–P2 amplitudes for predictable stimuli, while unpredictable stimuli elicited delayed compensatory responses^28^. In a later adaptation study, they observed stronger post-motor ERP activity over fronto-central areas during altered auditory feedback compared to unaltered feedback conditions, and this adaptation correlated with increased parietal ERP activity following vocalization onset, highlighting the neural dynamics of sensorimotor adjustments^29^.

Scheerer and Jones also used ERP-EEG during predictable and unpredictable pitch perturbation feedback to evaluate neural responses and their influence on subsequent vocal adjustments during speech production^33^. Using this same pitch-shifted auditory feedback paradigm, Chen et al. also examined how tone language experience influences neural correlates in native Mandarin and Cantonese speakers^30^. Similarly, Patel et al. used a training period with the pitch perturbation technique, showing that it can affect ERPs and voice pitch responses. These findings suggest brief training with pitch-shifted auditory feedback can modulate voice pitch regulation and potentially therapeutic applications^32^.

Cuadros et al. employed EEG to enhance temporal resolution and investigate the dynamics of the neurocomputational model DIVA. This was done within a paradigm of auditory feedback with first formant perturbation. They assessed key aspects of speech motor control, including sensorimotor integration and predictive coding^31^.

Building on these examples, further research utilizing EEG can continue to explore the complex interactions between auditory feedback and vocal motor control. By examining how the brain compensates and adjusts to changes in sensory feedback, these studies can provide deeper insights into the neural mechanisms underlying speech production. This understanding is crucial for developing advanced neurocomputational models and improving therapeutic interventions for speech disorders. However, most previous studies investigating sensorimotor integration in speech have focused on reflexive responses, such as rapid and involuntary vocal adjustments to unexpected auditory perturbations, most often in pitch or formant feedback. These transient responses stabilize vocal output momentarily^34–36^. In contrast, adaptive responses, like those elicited during the LE, arise from prolonged and systematic perturbations, such as sustained background noise, and involve ongoing adjustments in vocal behavior. Despite increasing interest in adaptive mechanisms, neurophysiological evidence in this domain remains limited, In particular, no study has directly examined how cortical networks support adaptive vocal adjustments during the LE, and EEG has not yet been applied to characterize its temporal dynamics.

**Fig. 1:**
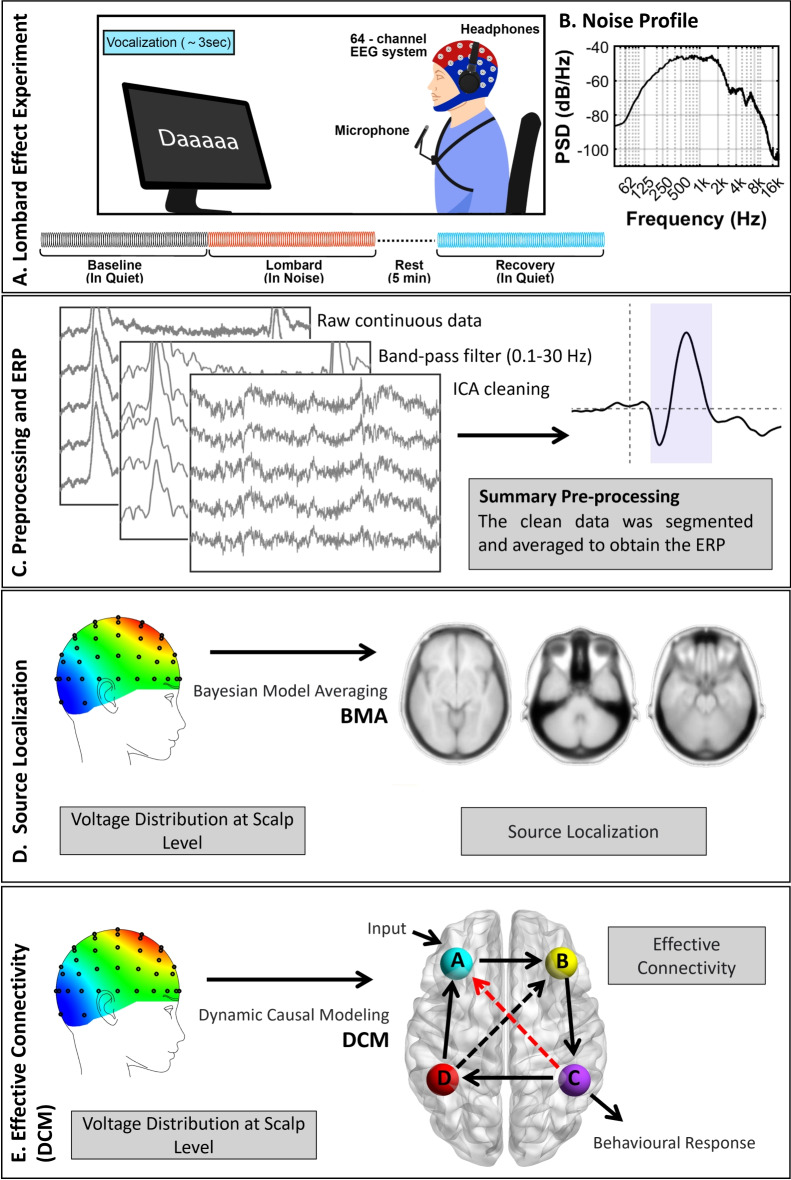
(**A**) An overview of the experimental design is shown. The experiment involved three conditions: Baseline (in quiet), Lombard (in noise), and Recovery (in quiet after five minutes of rest). (**B**) Noise Profile (Power Spectral Density of the Lombard Noise, 80 dB SPL). From C to E, the schematic diagram illustrates the analysis of electroencephalographic signals involved in the methodology. (**C**) A summary of the data preprocessing steps for obtaining the ERPs. (**D**) ERP source localization using BMA. (**E**) Effective connectivity analyses. Models were evaluated using DCM.

This study aimed to compare voice production, variations in electrophysiological neural activity, and dynamic relationships among brain regions in individuals with healthy voices under different acoustic backgrounds. We proposed three acoustic scenarios for the speakers: (1) in quiet environments, (2) under the LE (background noise), and (3) after five minutes of noise removal (Fig. 1). Based on previous findings^29^, we hypothesized a more significant behavioral and evoked response in the adaptive LE condition, specifically an increase in dB SPL and a significantly higher ERP in the N1-P2 complex components in LE compared to the quiet condition. The assessment of LE using EEG offers a unique opportunity to deepen our understanding of the neural underpinnings of vocal adaptation to background noise. By applying dynamic causal modeling (DCM), a generative approach for analyzing effective connectivity, this study examined how cortical networks dynamically interact to support adaptive vocal behavior, focusing on the interactions among regions associated with the N1–P2 complex during the LE.

## Results

We assessed variation in the acoustic measures and neurophysiological activity under three conditions: speaking in a quiet environment (Baseline condition), speaking under masking noise (Lombard condition), and speaking after 5 minutes of rest in a quiet environment (Recovery condition). These conditions were selected to explore how speaking in noise affects phonation and to describe the potential neurophysiological basis underlying the LE. All participants presented typical voices with no signs of voice disorder and had no reported hearing disorders.

### Acoustic measures

**Fig. 2:**
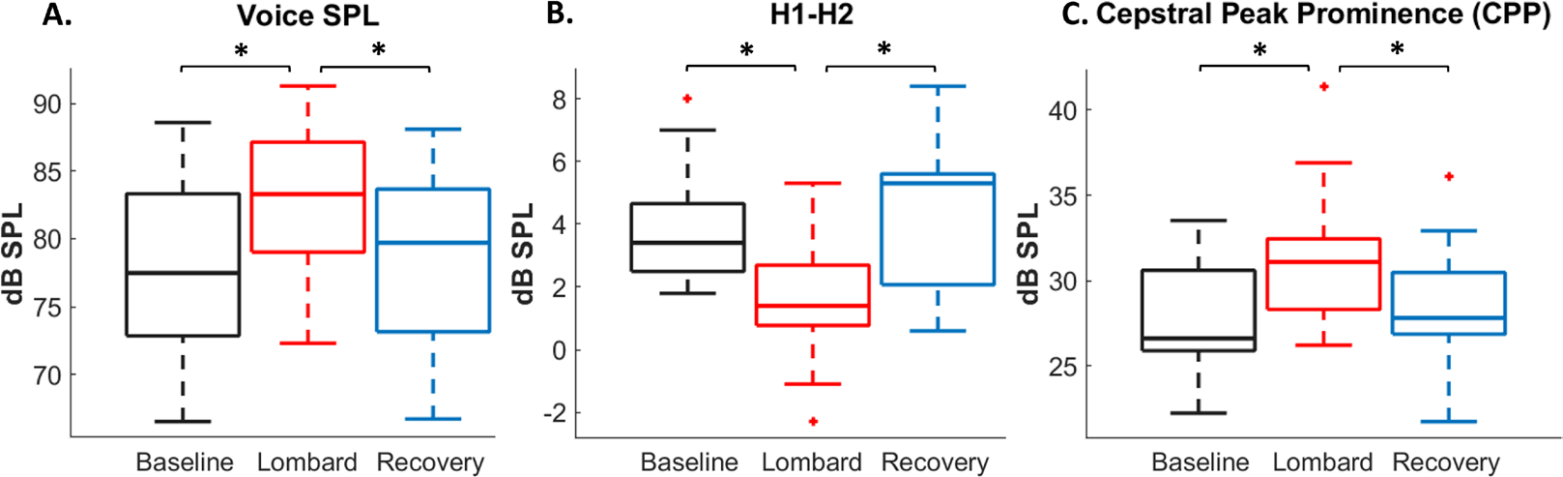
Results of the behavioral vocal response analysis across three experimental conditions: Baseline (black), Lombard in noise (red), and Recovery (blue). (**A**) Voice SPL, (**B**) H1–H2, and (**C**) CPP. Boxplots show the median and interquartile ranges. Asterisks (*) indicate significant differences between conditions (p < 0.05).

The LE was evident when participants spoke under masking noise. As a result, their SPL (Fig. 2a) increased significantly from the Baseline to the Lombard condition. SPL levels then decreased during the Recovery condition (quiet environment), five minutes after the noise was removed. A one-way repeated-measures ANOVA revealed a significant effect of condition on SPL ($F(2,40) = 44.210$, $p < 0.001, \eta ^2 = 0.689$). Post hoc tests with Bonferroni correction showed that SPL was significantly higher in the Lombard condition compared to both Baseline $(p < 0.001)$ and Recovery $(p < 0.001)$, with no significant difference between Baseline and Recovery $(p = 0.872)$.

This variation in SPL during speech in noise was accompanied by significant changes in H1-H2 (the difference in amplitude between the first and second harmonics; Fig. 2b) and Cepstral Peak Prominence (CPP; Fig. 2c). Both measures showed a significant main effect of condition (H1-H2: $F(2,40) = 28.107$, $p < 0.001, \eta ^2 = 0.584$; CPP: $F(2,40) = 10.121$, $p < 0.001, \eta ^2= 0.336)$. Bonferroni-corrected post hoc comparisons revealed significant differences between the Lombard condition and both Baseline and Recovery for H1-H2 $(p < 0.001)$, and similarly for CPP (Lombard vs Basal:$p < 0.001$; Lombard vs Recovery $p = 0.007$). No significant differences were observed between Baseline and Recovery for either measure $(H1-H2: p=0.104; CPP: p=0.216)$.

### Electrophysiological measures

**Fig. 3:**
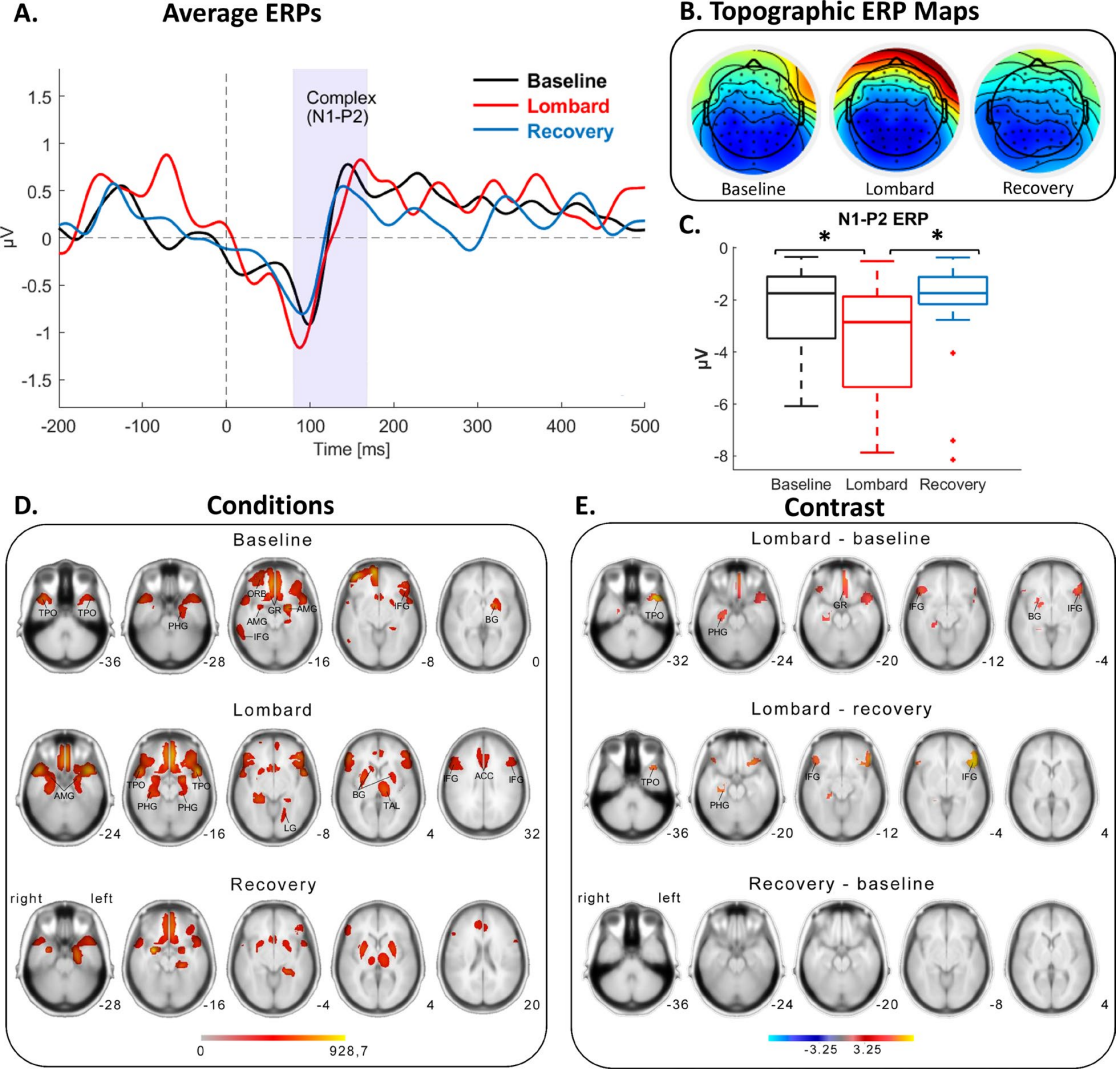
(**A**) Grand mean ERPs in all subjects at a pooling of electrodes (average of O1, O2, Oz, P1, P10, P2, P3, P4, P5, P6, P7, P8, P9, PO3, PO4, PO7, PO8, POz, and Pz). (**B**) Topographic distribution maps for each condition on a scalp map of 64 EEG electrodes. (**C**) Mean N1–P2 ERP amplitude for each condition. (**D**) Source localization for each condition. (E) Representation of the statistical analysis of the source localization for the contrasts between Lombard-Baseline, Lombard-Recovery, and Recovery-Baseline.

The mean ERPs for each condition and the topographic distribution maps are at the top of Fig. 3. The statistical analysis results indicated a significant effect among the conditions $(F(2,40)=7.943, p=0.001, \eta ^2=0.284)$. Post hoc tests using Bonferroni’s correction revealed that the amplitude of the N1-P2 complex was significantly higher for the Lombard condition compared to Baseline $(p=0.009)$ and Recovery $(p=0.002)$. However, the amplitude of the N1-P2 complex did not differ significantly between Baseline and Recovery conditions $(p=0.62)$(Fig. 3a–c).

The results of the second experiment showed that the N1–P2 complex elicited during passive listening to speech in noise followed a different pattern across conditions compared to the active task. Specifically, N1–P2 amplitudes were reduced in the Lombard condition relative to both Baseline and Recovery, while Baseline and Recovery remained comparable to each other (see Supplementary Information for details).

Source localization analyses of the N1-P2 complex of the ERP elicited by participants’ own voices revealed distributed brain activation within the frontal, limbic, and temporal lobes (Fig. 3d). The topographic distribution of the N1-P2 generators was consistent across conditions (Baseline, Lombard, and Recovery). It included brain areas that are i) crucial for speech comprehension, ii) relevant for the processing of the auditory feedback of one’s own voice, and iii) activated due to listening efforts in noisy environments. In the frontal lobe, the N1-P2 generators included the orbitofrontal gyrus (ORB), the gyrus rectus (GR), and the inferior frontal gyrus (IFG). In the limbic lobe, sources of the ERP were estimated in the hippocampus/parahippocampal gyrus (PHG) and the amygdala (AMG). Lastly, temporal areas associated with the N1-P2 generation included the temporal pole (TPO) and the inferior temporal gyrus (ITG).

In comparison with the brain activity elicited by the auditory feedback of one’s own voices in quiet (Baseline), self-monitoring of speech in noise (Lombard) was associated with higher activations in the IFG, the TPO, the GR, the PHG, and the basal ganglia (BG) (voxel-wise t-test: $p \le 0.05$, corrected for multiple comparisons using permutations test, 5000 randomizations) (Fig. 3e). The same areas, except for the GR and the BG, also displayed hyperactivation during the LE when compared to the brain activity estimated in the Recovery condition (voxel-wise t-test: $p \le 0.05$, corrected for multiple comparisons using permutations test, 5000 randomizations) (Fig. 3e). Cortical hypoactivation was not associated with self-monitoring of speech in noise in either of these contrasts (voxel-wise t-test: $p \ge 0.05$). Likewise, the brain activation did not significantly differ between the Baseline and the Recovery conditions (voxel-wise t-test: $p \ge 0.05$) (Fig. 3e).

### Effective connectivity

Three DCM models, which differed in the specification of forward and modulatory connections among A1, TPO, PHG, and IFG, were inverted for each subject’s evoked responses of the LE. Considering the source locations of our data, the included regions were A1$\left[ 50, -10, 8\right]$, TPO$\left[ 55, 10, -15\right]$, PHG$\left[ 30, -20, -15\right]$, and IFG$\left[ 50, 30, 4\right]$.

**Fig. 4:**
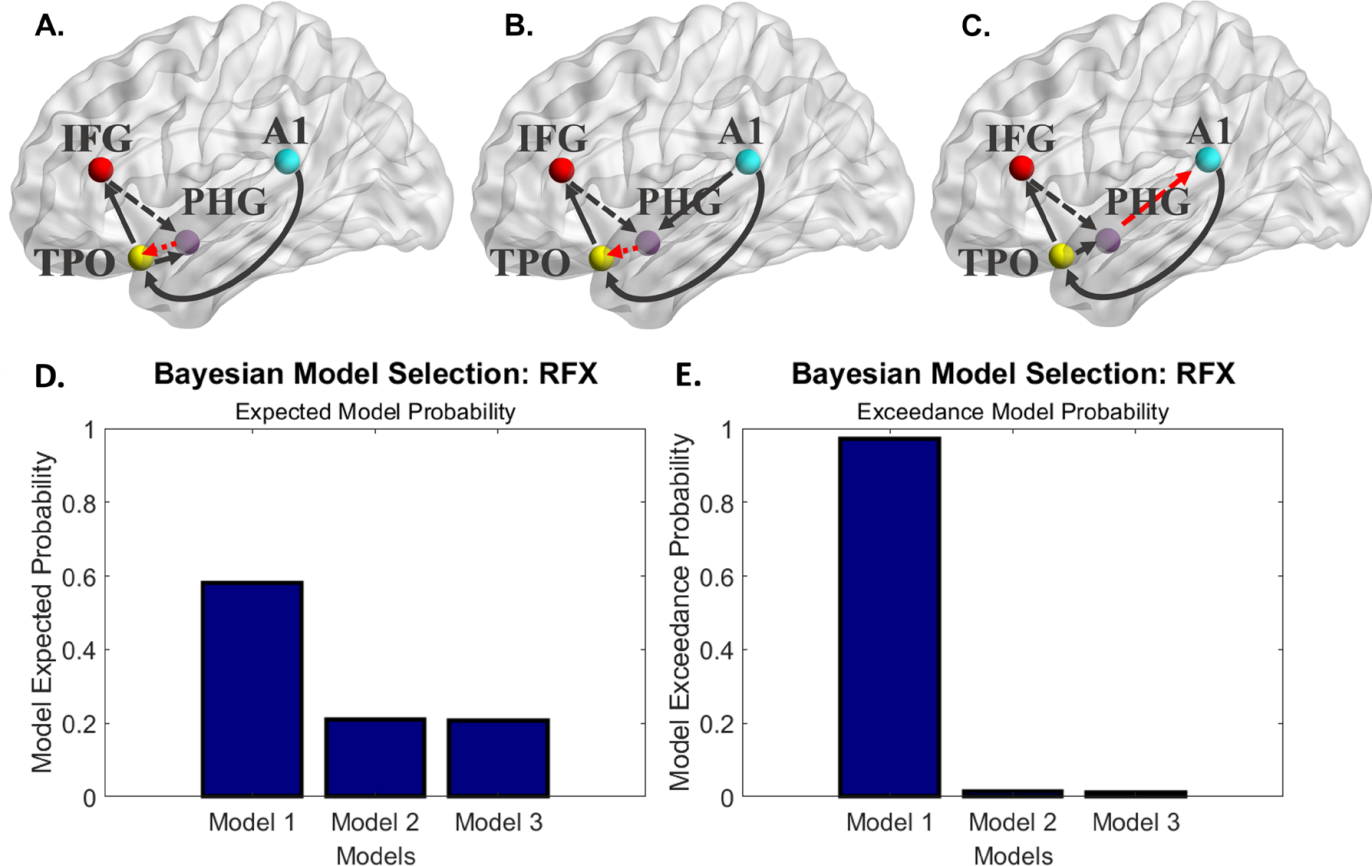
Three different DCMs were constructed based on the BMA source localization analysis, incorporating A1, TPO, PHG, and IFG. (**A**–**C**) Candidate models of effective connectivity (see Results for details). Bayesian model selection (RFX) results are displayed as (**D**) expected model probabilities and (**E**) exceedance probabilities across subjects. Model connectivity was visualized using BrainNet Viewer^103^.

Specifically, in Model 1 (Fig. 4a), the input was generated in A1; forward connections were specified from A1 to TPO, from TPO to IFG, and from TPO to PHG. Additionally, a backward connection from IFG to PHG was specified, and a modulatory connection was outlined from PHG to TPO. In Model 2 (Fig. 4b), the only modified forward connection was TPO to PHG, which A1 shifted to PHG. In Model 3 (Fig. 4c), the change was specified in the modulatory connection from PHG to A1, excluding TPO from modulation.

Bayesian model selection (BMS) analysis using the random effects (RFX) inference method, at the group level, indicated exceedance probabilities of 0.9716 (97.16%), 0.0152 (1.52%), and 0.0132 (1.32%) for Models 1, 2, and 3, respectively. Therefore, the likelihood of Model 1 was higher than that of Models 2 and 3. The three contrasted models included an equal number of forward, backward, and modulatory connections. Consequently, these results cannot be explained by a more complex model.

## Discussion

Our results showed that participants generated an adaptive response to speaking under noise (Lombard condition) characterized by an increase in a mean of 5.2 dB in their voice intensity (SPL) compared to speaking in quiet (Baseline condition). This variation is consistent with previous LE studies that used masking noise with higher spectral energy in the speech frequency range (100–5000 Hz, 75–90 dB)^8,9,11,37,38^. Also, the mean variation in SPL is similar to that reported in previous studies when the speakers generated voluntarily loud phonatory tasks^39–42^. Participants also showed a gradual change in SPL across trials during the Lombard condition, consistent with an adaptive adjustment to sustained background noise^43^ (see Supplementary Materials). After 5 minutes of rest, the participants return to intensity levels close to those observed in the Baseline condition, This finding is concordant with previous studies that included a recovery stage after speaking in noise to examine the LE in speakers with typical voices^37^.

In addition to the increase in SPL, H1-H2 decreases under masking noise conditions. Previous studies have reported that this measure is related to voice quality. When H1-H2 decreases, the voice is perceived as having characteristics typical of a pressed phonation, including a more abrupt closing phase of the glottal cycle^44^. This variation may be attributed to the increased velocity of the vocal fold closing phase due to the LE. As a result, the voice quality during speech in noise shifts toward a more pressed configuration and returns to Baseline after five minutes of rest. These vocal adjustments are consistent with patterns of vocal hyperfunction, characterized by increased effort and glottal tension^45,46^.

Regarding the CPP measure, our results showed an increase in the Lombard condition and a decrease in the Recovery condition. CPP is a measure of voice quality that reflects the strength of the harmonic structure in the voice signal. In our data, this means that the harmonic components were stronger during the Lombard condition, leading to improved voice quality that likely contributes to greater intelligibility in noise. This variation in CPP could also be associated with modifications in the spectral components of speech, which reinforce the harmonic component between 2 kHz and 5 kHz, as reported in previous studies^3,47^.

From a neurophysiological perspective, the analysis of ERP responses revealed variations among conditions, with an increase in the N1-P2 complex when participants spoke in noisy environments, in contrast to the Baseline and Recovery conditions. The heightened N1-P2 complex during the Lombard condition may be associated with an adaptive response to the LE. This variation in N1-P2 is similar to the findings of Behroozmand and Sangtian using a pitch shift reflex paradigm, suggesting that the response generated as a result of the perturbation, particularly the post-motor response between 0–200 ms, may be involved in vocal sensorimotor adaptation^29^. Although we did not observe significant associations between acoustic adjustments and N1–P2 amplitude (see Supplementary Materials), the ERP response was consistent across participants and showed a large effect, supporting the main conclusions of the study.

The passive-listening experiment provides additional insight into this effect. In this secondary task, N1–P2 amplitudes were reduced in the Lombard condition relative to Baseline and Recovery, contrasting with the pattern observed during active speech production, where the Lombard condition elicited larger N1–P2 amplitudes. Overall ERP latencies and maximum amplitudes also differed between the active and passive experiments.

The LE is present in many vertebrates, such as fish, birds, and mammals. Recent theoretical frameworks suggest that the LE is strongly related to subcortical areas, including structures such as the AMG, and hypothalamus. These areas trigger the motor compensatory response of speech muscles during the LE to maintain oral communication^12^. Studies in non-human primates suggest that the brain cortex may serve as a modulating system for subcortical pathways to regulate the degree of motor control compensation for speech and voice muscles. Notably, these studies suggested that LE is potentially modulated by large cortical networks comprising sensorimotor systems, including visual, primary motor, somatosensory, and prefrontal cortical areas^12,23,48,49^. In humans, fMRI studies suggest that speaking in noisy environments could be associated with motor, somatosensory, and prefrontal cortical activation. In these pathways, the cortical regions could play an essential role in modulating the response of the LE^11,23^.

Our results using BMA showed activation of cortical areas involved in voice monitoring (ORB, IFG, TPO, and ITG). During the Lombard condition, bilateral activation increased in the IFG, TPO, GR, PHG, and AMG compared to Baseline. When comparing Lombard and Recovery, greater activation in IFG, TPO, and PHG was observed in the Lombard condition relative to Recovery, indicating that these cortical areas play a modulatory role in the LE.

Computational models of speech production propose that the IFG plays a role in feedforward control and auditory feedback error monitoring^15,19,21,50^. Consistent with this, EEG and fMRI studies using pitch-shift and formant-shift paradigms have reported IFG activation during auditory feedback error detection and correction^19,51,52^.

Similar findings have been observed using high-definition transcranial alternating current stimulation (HD-tACS), showing that the left IFG plays a critical role in the feedback control network that mediates vocal compensation^53^. Notably, pitch-shift studies and computational models often report right-lateralized IFG activation, whereas our data showed bilateral activation during the Lombard condition. This suggests that pitch-shift error processing does not fully overlap with the cortical mechanisms engaged during speech in noise in the LE.

Focusing on the specific role of the IFG in the LE, Meekings reported variations in BOLD signal in this region during a speech-in-noise paradigm^11^. Additionally, that study showed increased activation in the bilateral STG during speech production under continuous noise^11,22^. However, our results did not indicate involvement of the STG. These differences could be associated with the distinct approaches used to study the LE. In our case, the use of EEG provides higher temporal resolution, although it is limited in spatial resolution, which may contribute to differences in the spatial activation patterns observed across modalities.

Another area with increased activity during speaking in noise in our results is TPO. Previous studies have associated this region with the processing of auditory information, auditory memory, and auditory multimodal integration^54,55^. Interestingly, recent studies in primates propose that this area has an important role in the connectivity with other areas related to motor and auditory processing^55^. Furthermore, studies using fMRI signals found an increase in BOLD signal associated with TPO when the participants recognized speech-in–noise compared to clear speech^26^.

Our results showed increased activation in the hippocampus/PHG and AMG during the LE. Animal studies propose that these medial temporal lobe structures and subcortical components are involved in voice and speech motor control^56^. Moreover, Luo suggests that these areas contribute to the LE response by supporting motor corrections to noise, modulated by prefrontal cortical regions^12^. PHG has been implicated in speech production, verbal communication, and speech monitoring^57–66^. In this context, the masking noise could be interpreted as an unexpected event that triggered increased PHG activity. Moreover, hippocampal activity has been linked to mismatch detection during unexpected events^67^. Structural MRI studies further indicate that increased local volumes in PHG and right AMG predict larger vocal compensations to pitch perturbations^68^. Together, these findings suggest that PHG and AMG activation in our study may reflect enhanced auditory–motor integration mechanisms under the LE.

We next used DCM to investigate the effective connectivity involved in sensorimotor adaptation during speech in noise. The findings indicate that forward, backward, and modulatory connections can explain the differences between the evoked responses in the Lombard and Baseline conditions.

The best-fit connectivity model (M1) proposes a forward network operating across distinct cortical hierarchy levels. This network originates from A1 and establishes connections with TPO. From TPO, two distinct connections project toward the IFG and PHG (Fig. 4). Interactions within the forward network may relate to the construction and transmission of sensory information^69^. Within this framework, motor schemas are proposed to account for predicted sensory consequences. Consistent with this prediction process, our model includes a backward coupling between the IFG and PHG (see Fig. 4).

In line with prior findings, these areas have been recognized as crucial components of the cortical network for vocalization control^52,66,70^. Specifically, the IFG has been associated with motor planning^71^, showing increased activation during attentional mechanisms involved in auditory masking^11,14^. The PHG, beyond its traditional association with memory, has also been implicated in predictive processes in speech production based on contextually stored representations^66,68^.

In addition, our best-fit model incorporates a backward connection from the PHG to the TPO, which exerts a modulatory influence (Fig. 4a). We suggest that this pathway represents an auditory feedback connection that monitors both the generated prediction (IFG-PHG) and the current auditory state in TPO. These top-down connections emphasize processes of feedback and integration, consistent with prior evidence highlighting the role of anterior temporal regions in feedback processing^11,72^. The modulatory influence of this connection may therefore contribute to the generation of an adaptive listening response in noisy environments^69^.

Several limitations should be considered. First, the pre-stimulus ERP interval was not completely flat. We do not attribute this effect to the experimental timing. The minimum interstimulus interval was two seconds, reducing the possibility that the baseline was contaminated by neural activity from the preceding trial. Instead, this effect is likely related to the nature of the task, which involves overt speech production and therefore includes speech-related muscle activity that may introduce some distortion in the ERP signal^73,74^. In addition, the second ICA step applied during processing may also have contributed to the observed baseline deviation. Future studies could further examine how this step influences ERP baseline stability in speech-production paradigms.

Second, the fixed order of conditions in both active and passive experiments makes it difficult to determine whether the results are driven by the LE or time-dependent influences such as fatigue or habituation. In addition, passive listening to one’s voice does not fully reproduce the perceptual and sensorimotor context of speaking, which may explain differences in overall ERP latency and maximum amplitude between experiments. However, the complementary passive listening experiment was not designed to directly match the ERP morphology of the active task, but rather to provide a control condition to assess whether the effects observed in the main experiment could be explained solely by condition order or exposure to background noise.

Third, although BMA includes anatomical constraints that help constrain deeper source estimation, the spatial resolution of a 64-channel EEG montage inherently limits the precision with which regions such as the PHG and AMG can be localized. Thus, while the overall activation pattern aligns with previous findings^66,68^, the exact spatial specificity of these estimates should be interpreted with caution.

The BMA approach used to estimate the generators of the ERP effectively mitigates the depth-related bias that characterizes many conventional EEG inverse solution methods, whereby deep sources are systematically underestimated in favor of more superficial ones. This bias typically leads to solutions that preferentially attribute the observed data to generators located near the sensors, while deep generators are either omitted or inaccurately reconstructed. BMA has been shown to recover the spatial localization of both cortical and subcortical sources with reasonable and consistent accuracy. Likewise, EEG source estimations using BMA are robust to variations in the number of sensors. Nevertheless, activity estimated in subcortical regions such as the PHG and AMG should be interpreted as reflecting engagement of broader limbic–temporal networks, rather than as definitive evidence for highly focal generators within specific subcortical substructures^75,76^.

From a methodological standpoint, BMA provides an internal mechanism for balancing data fit and model complexity through the use of Bayesian model evidence. In our analysis, individual source models are weighted by their posterior model probabilities and then averaged, which reduces overfitting by penalizing overly complex models that do not substantially improve the explanation of the data. However, we did not compute additional out-of-sample goodness-of-fit indices or cross-validated performance metrics (e.g., variance explained on held-out trials or sessions). This absence of explicit cross-validation constitutes an additional limitation of the present study. Future work should complement the Bayesian model evidence with cross-validated predictive measures and, ideally, with convergent data from higher-density EEG, MEG, or structural/functional MRI, to more rigorously assess the reliability and anatomical specificity of deep source estimates.

Finally, because DCM relies on the same source estimates used in the localization analysis, some uncertainty in deeper sources (such as PHG or AMG) can also affect the connectivity results. Accordingly, the DCM results should be viewed as describing plausible large-scale interactions supported by the data within the scope of the present methodology.

In conclusion, the LE engages a coordinated neural response characterized by increased vocal intensity and enhanced N1–P2 amplitudes, reflecting heightened auditory–motor integration. Source localization further identified distributed activation across frontal and temporal regions, including medial temporal and subcortical structures, suggesting the recruitment of areas supporting predictive and feedback processes. Building on these findings, our DCM inversion results provide insight into the effective connectivity underlying these adaptations. The identified forward, backward, and modulatory influences highlight dynamic interactions among temporal and frontal regions that support flexible vocal adjustments in response to background noise. Together, these results contribute to a deeper understanding of the neurophysiological mechanisms underlying adaptive speech behavior in challenging acoustic environments.

## Methods

### Participants and procedure

Twenty-one volunteers were recruited for this study, including 9 females and 12 males $(mean\ age = 25.8, SD= 5.06)$. This sample size was based on previous ERP/EEG studies using comparable auditory feedback perturbation paradigms and experimental designs^32,77–79^. All participants were right-handed and native Spanish speakers. As part of the screening procedure, a speech-language pathologist evaluated each participant based on case history, clinical evaluation, aerodynamic and acoustic measures of vocal function, and a Consensus Auditory-Perceptual Evaluation of Voice^80^. Participants showed typical voice quality with no signs of vocal pathology and did not report a history of speech, language, hearing disorders or other neurological disorders. Volunteers with either theoretical or practical vocal training were excluded, including those with experience in singing or speech-language pathology. In addition, participants passed a pure-tone hearing screening, which required positive responses to air-conduction stimuli in both ears at 20 dB HL at octave frequencies between 250 Hz and 8000 Hz using a clinical audiometer (Model AD629, Interacoustics A/S, Middelfart, Denmark).

All volunteers gave written informed consent, and the procedure was approved by the Research and Ethics Committee of the Faculty of Medicine, Universidad de Valparaíso, Chile (statement code 52015), in accordance with national regulations for human research and the Declaration of Helsinki.

The experiment was composed of three sequential acoustic background conditions: Baseline (in quiet), Lombard (in noise), and Recovery (in quiet after five minutes of rest). The conditions were presented in a fixed sequence because the paradigm was designed to examine an adaptive transition from quiet speech to speech produced in background noise, followed by post-noise recovery. This structure requires beginning in a quiet condition to establish a valid baseline reference for subsequent comparisons. Counterbalancing the order would interrupt the transition from quiet speech to noise-driven adaptation and subsequent recovery. Therefore, the sequential structure of the task reflects the temporal dynamics of the LE and follows procedures used in adaptive paradigms^29^.

In each condition, participants were asked to utter eighty syllables at a comfortable pitch and loudness. The syllables were /*pa*/, /*da*/, /*ta*/, and /*ba*/, and they were presented randomly. Each syllable was presented for two seconds by visual cues displayed on a screen (Fig. 1a). Participants were instructed to speak at a comfortable loudness level and maintain the vocalization for the same amount of time as the stimulus, while receiving real-time auditory feedback of their own voice through headphones. The experiment included a 2-second intertrial interval, and every five stimuli the intertrial interval was extended to 8 seconds, resulting in about 8 minutes per condition. The voice and EEG signals were recorded in each condition. Using vowels and syllables enabled reliable and reproducible ERP responses to be obtained.

In the Lombard condition, speech-shaped noise at 80 dB SPL was generated using a clinical audiometer (Model AD629, Interacoustics A/S) and presented through closed headphones (Model 7510, RadioEar), which are commonly used in standardized clinical speech-in-noise tests. The noise showed increased spectral energy around 250 Hz and 2000 Hz (Fig. 1b). This level is comparable to those used in previous Lombard studies^4,5,24^ and is sufficiently loud to elicit a robust LE while avoiding hearing discomfort or vocal/auditory fatigue^81^. Due to the closed-ear design of the headphones, a certain degree of attenuation of the participants’ voices could occur. However, these headphones have previously shown no significant effect on speech acoustics^5^.

The Recovery condition was included to explore the potential persistence of the LE after speaking under noise. A five-minute rest between the Lombard and Recovery conditions was selected to allow participants to recover from possible vocal loading effects, as suggested in previous studies^82,83^.

The five minute rest period was selected based on evidence showing that aerodynamic and acoustic measures, such as phonation threshold pressure and pitch, show significant recovery within 5 minutes after vocal loading^83^. Although the precise time course of neural recovery is less well established, studies in speech production and related motor tasks indicate that short breaks are sufficient to reset task-related states, supporting the use of a brief rest period in this paradigm^84^.

To determine whether ERP amplitudes during active speech production could be explained by auditory perception of masking noise or by the fixed order of conditions, we conducted a second experiment aimed at investigating cortical responses during passive listening. In this experiment, twenty participants listened to a voice recording through headphones that included the same sequential structure as the active task: speech in quiet, speech in noise, a 5-minute resting period, and speech in quiet again (Baseline, Lombard, and Recovery). Detailed information on participants and procedures is provided in the supplementary information.

### Acoustic data acquisition

The acoustic signal was obtained using a microphone (B&K, model 4961; Nærum, Denmark) located in front of the participant at 15 cm from the lips at a 45-degree offset in the axial direction and amplified by a B&K 1705 signal conditioner. The acoustic signal was calibrated in dB (re 20 $\upmu$Pa) using a Larson Davis calibrator (model CAL200, Depew, NY, USA). Next, signals were sampled at 20 kHz with 16-bit quantization and low-pass filtered (3 dB cutoff frequency of 8 kHz) using a National Instrument DAQ model USB-6363 BNC.

### Electrophysiological data acquisition and ERP pre-processing

The EEG signals were recorded using a BioSemi ActiveTwo system with 64 active electrodes at a sampling rate of 4096 Hz. The location of each active Ag/AgCl electrode was according to the standard 10–20 montage. The EEG data was synchronized with the acoustic signal. The continuous EEG data was pre-processed offline using Brain Vision Analyzer 2.0® software (Brain Products GmbH, Munich, Germany) following standard procedures for ERP computation. Recordings were first band-pass filtered among 0.1- 30 Hz with a zero-phase shift Butterworth filter of order 8. Then, the sample rate was decreased to 512 Hz. Ocular, muscular, and line noise artifacts were corrected and removed by visual inspection with the Independent Component Analysis (ICA) method^85^, which follows Chaumon et al.’s approach. Across participants, between two and five components were typically removed, while six to seven were excluded only in a few cases. Data were re-referenced to the average of all channels, and epochs were segmented from -200 to 500 ms around the onset of vocalization. Epochs were rejected if the amplitudes exceeded a maximum voltage of ± 50 $\upmu$V. Epoch exclusions were consistently below 5% of the 80 epochs per condition, with no exclusions for most participants. In the epochs average, baseline correction was performed using a 200 ms prestimulus window aligned to the onset of vocalization.

After baseline correction, an additional ICA-based decomposition step was applied to the averaged epoched data. ICA of averaged ERP waveforms has been used in previous studies to separate residual non-neural activity that is not consistently time- or phase-locked to the stimulus^86^. Previous work has shown that ICA-based approaches can be used to extract residual artefactual activity and improve the interpretability of ERP components^87,88^, particularly in datasets with low signal-to-noise ratio, such as EEG recordings from children where ERP components are difficult to detect^89^. In addition, ICA can decompose ERP responses into temporally overlapping neural processes and isolate physiologically meaningful components^86,90–93^. In the present LE paradigm, the ICA step in our analysis was used to identify residual non-neural activity that remained after standard preprocessing. Although muscular artefacts were controlled during preprocessing, overt speech production involves jaw movements and facial muscle activity that can introduce noise affecting the identification of ERP components^74,94^.

Following Yang et al., the components obtained from the additional ICA decomposition were evaluated based on their temporal and spatial stability^88^. Regarding the temporal aspect, components whose peak latencies fell within the expected time window of the underlying neural activity, such as the N1 and P2 peaks, were retained. With respect to spatial criteria, components exhibiting a dipolar scalp topography consistent with cortical generators were preserved, whereas components showing scattered or non-physiological topographies were considered artefactual activity and excluded from the reconstruction. Thus, rather than redefining ERP components, the ICA decomposition was used to isolate latent neural processes contributing to the ERP waveform while removing residual non-neural activity. Across participants, one of the four extracted components was typically removed and the ERP signal was reconstructed from the remaining components. Importantly, the overall ERP patterns were qualitatively similar when the second ICA step was omitted, with comparable N1 and P2 amplitudes across conditions, suggesting that the conclusions do not depend on this processing step (see Supplementary Fig. S2).

This second ICA step resulted in the ERP baseline not always being centered at zero. Residual muscle activity from speech production may further contribute to baseline instability^73,74^. For these reasons, we focused on the N1–P2 peak-to-peak amplitude rather than on the absolute amplitudes of the individual components, which is a standard approach in ERP research^27^.

### ERP source localization

Source localization was estimated using the Bayesian Model Averaging (BMA)^75^, based on a standard anatomical model. BMA calculates the brain sources considering anatomical constraints to solve the EEG/MEG inverse problem. Thus, it favors/ penalizes brain regions based on the probability of contributing to data generation. Moreover, it allows for the estimation of deep EEG generators^76^.

Cortical activation maps were calculated for each scalp voltage distribution based on the latencies of the N1-P2 components of the ERP, obtained as the average across electrodes O1, O2, Oz, P1, P10, P2, P3, P4, P5, P6, P7, P8, P9, PO3, PO4, PO7, PO8, POz, and Pz. For each participant and condition (Baseline, Lombard, Recovery), source estimates were averaged within a ±10 ms window centered on the individual N1–P2 peak latency, to ensure comparable time sampling across participants.

### Effective connectivity

To investigate the evoked response observed in the LE at the network level, we used DCM^95,96^. We determined a target time window between 1 and 400 ms since this signal captures the neural responses for the ERP N1-P2 complex. To assess the impact of conditions on connectivity, the basal condition was taken as a reference, while the Lombard condition was considered the signal of interest. The effective connectivity analysis - modeled as equivalent current dipoles (ECDs) - was based on the source location of our data obtained through BMA software, with coordinates aligned to the MNI head standard for each source. We generated three connectivity models using the selected regions, each detailing different specifications for forward, backward, and modulated connections.

Subsequently, Bayesian model selection (BMS) was applied to determine the model that accurately fits the data. The comparison of models utilizes logarithmic evidence under the free energy criterion. This criterion consists of two elements: the precision (fit) term, obtained from the log-likelihood of the data, and the complexity term^97^. The group-level analysis employed the random effects (RFX) inference method, which accommodates the possibility that the best model may vary among participants and assumes homogeneous intra-subject variance across the entire population.

### Statistical analysis

From the acoustic signal, the vocal SPL was computed for each vocalization using a window size of $\sim$200 ms. For testing our hypotheses, repeated measures analyses of variance (ANOVA) were performed to analyze the changes in the acoustic SPL measures. A Post hoc test using Bonferroni’s correction was realized to test the statistical significance between conditions ($\alpha = 0.05$). In addition, the acoustic parameters H1–H2 and CPP were extracted from the vocalizations using standard procedures^98,99^. H1–H2 was defined as the difference in log-magnitude between the first and second harmonic, computed from the power spectrum^46^. Cepstral Peak Prominence (CPP) corresponds to the magnitude of the highest peak in the power cepstrum^100^. Both measures were estimated using a custom MATLAB script (MathWorks, version 2020a) with a 200-ms Hamming window applied to each vocalization, without smoothing. A total of 240 tokens per participant were analyzed across all conditions.

The statistical analysis of the ERP responses was performed in a 2-point average window around the peak of the N1 and P2 components concerning the onset of vocalization. Subtraction of these values was performed to analyze the variation in the N1-P2 complex. N1 and P2 peaks were identified using the peak-detection tool in Brain Vision Analyzer, which searches for the maximum amplitude within predefined latency windows for each component. After the automatic detection, all peaks were visually inspected for every participant and condition to ensure accurate identification. A repeated measures ANOVA test was used, including the conditions of N1-P2 Baseline, N1-P2 Lombard, and N1-P2 Recovery, for the electrodes grouped in a position of interest (O1, O2, Oz, P1, P10, P2, P3, P4, P5, P6, P7, P8, P9, PO3, PO4, PO7, PO8, POz, Pz). Electrode selection was based on the scalp locations that exhibited the most robust and consistent N1–P2 topography in our recordings, using a data-driven approach.

For all acoustic and ERP analyses, assumptions of normality and sphericity were evaluated through Shapiro–Wilk tests and Mauchly’s test, respectively. All ANOVA assumptions were met.

The statistical analysis of the ERP cortical activity maps was performed using the standardized low-resolution brain electromagnetic tomography (sLORETA) software^101^, which was employed exclusively for statistical testing and not for source estimation. To increase the stability of the permutation analysis, we selected the 2000 voxels with the strongest absolute activity from the full BMA output of 21 926 voxels, separately for each contrast (Lombard vs Baseline, Lombard vs Recovery, and Recovery vs Baseline). For each contrast, we calculated the voxel-wise within-subject differences and obtained paired t-values using unequal-variance estimates. The condition labels were shuffled within each participant, and 5000 permutations were run to obtain the empirical distribution used for voxel-level multiple-comparison correction. No transformations or normalization steps were applied. The resulting significance maps were thresholded using the permutation-derived critical t-value (p < 0.05, corrected).

In the DCM analysis, the exceedance probability resulting from the RFX methods for BMS is employed to express confidence in the posterior probability that provides the best explanation for the data. This variable sums up to 1 across all tested models; therefore, a value above 95% indicates a significant outcome^102^.

## Supplementary Information

Supplementary Information.

## Data Availability

The datasets generated during and/or analyzed during the current study are available from the corresponding author on reasonable request.
