# Supplementary material for: Neural dynamics and acoustic adaptations during the Lombard effect: evidence from EEG and dynamic causal modeling
Source: Sci Rep. 2026 Apr 24;16:18939. doi: 10.1038/s41598-026-49995-x (PMC13276361; doi:10.1038/s41598-026-49995-x)
Supplement: Supplementary file 1 — Supplementary Information. [file 41598_2026_49995_MOESM1_ESM.pdf]

Supplementary Information

Supplementary Experiment: Passive listening control

This supplementary experiment was designed as a control to further validate the interpretation of the ERP results from the main study. To determine whether the increased amplitude of the N1-P2 complex was specifically elicited by the LE or as a result of listening to speech in background noise, we conducted a second experiment involving passive listening. A separate group of twenty-one participants ($mean\ age = 25.05, SD = 4.4$), consisting of 11 males and 10 females, different from those who took part in the main experiment, completed a passive listening task. All participants were right-handed, native Spanish speakers, and exhibited typical voices with no signs of voice disorders. They also reported no history of speech, language, hearing, or neurological disorders, and none had theoretical or practical vocal training, including experience in singing or speech-language pathology. Hearing abilities were confirmed through a pure-tone screening, which required positive responses to air-conduction stimuli presented at 20 dB HL in both ears across octave frequencies from 250 Hz to 8000 Hz, using a clinical audiometer (Model AD629, Interacoustics A/S, Middelfart, Denmark). EEG signals were recorded using a BioSemi ActiveTwo system, following the same electrode setup and configuration used in the main experiment.

The experiment consisted of three sequential acoustic background conditions identical to those used in the main experiment: Baseline (in quiet), Lombard (in noise), and Recovery (in quiet after five minutes of rest). The key difference was that participants did not vocalize but instead passively listened to pre-recorded vocalizations. A female voice recording was used for female participants, and a male voice recording for male participants. These recordings included the same syllables used in the main experiment: $/pa/$, $/da/$, $/ta/$, and $/ba/$. Each vocalization had an approximate duration of 3 seconds. All volunteers provided written informed consent in accordance with the Declaration of Helsinki. The study was approved by the Research and Ethics Committee of the Faculty of Medicine at the Universidad de Valparaíso, Chile. Data analyses were conducted following the same procedures described in the Methods section of the main article. However, a second ICA step was not applied in this dataset due to the absence of vocalization-related artifacts.

A one-way repeated-measures analysis of variance (ANOVA) was conducted to compare the mean amplitude of the N1-P2 complex across the three experimental conditions: Baseline, Lombard, and Recovery. Using an alpha level of 0.05, the test revealed a statistically significant effect ($F = 10.909, p = 0.001, \eta^2 = 0.353$). Post-hoc Bonferroni tests indicated that the mean amplitude in the Baseline condition was significantly different from that in the Lombard condition ($p < 0.001$). Similarly, a significant difference was found between the Lombard and Recovery conditions (p = 0.001). However, no significant difference was observed between the Baseline and Recovery conditions ($p = 0.533$).

Specifically, a decrease in the amplitude of the N1-P2 complex was observed during passive listening in the Lombard condition. In contrast, the Baseline and Recovery conditions showed greater and comparable amplitudes.

Figure S1 shows the ERP waveforms for each condition. The contrast between the active and passive lombard experiments suggests that the increased N1-P2 amplitude observed in the main experiment cannot be explained solely by auditory masking. This reinforces the interpretation that the effect reflects neurophysiological activity specifically elicited by the LE.

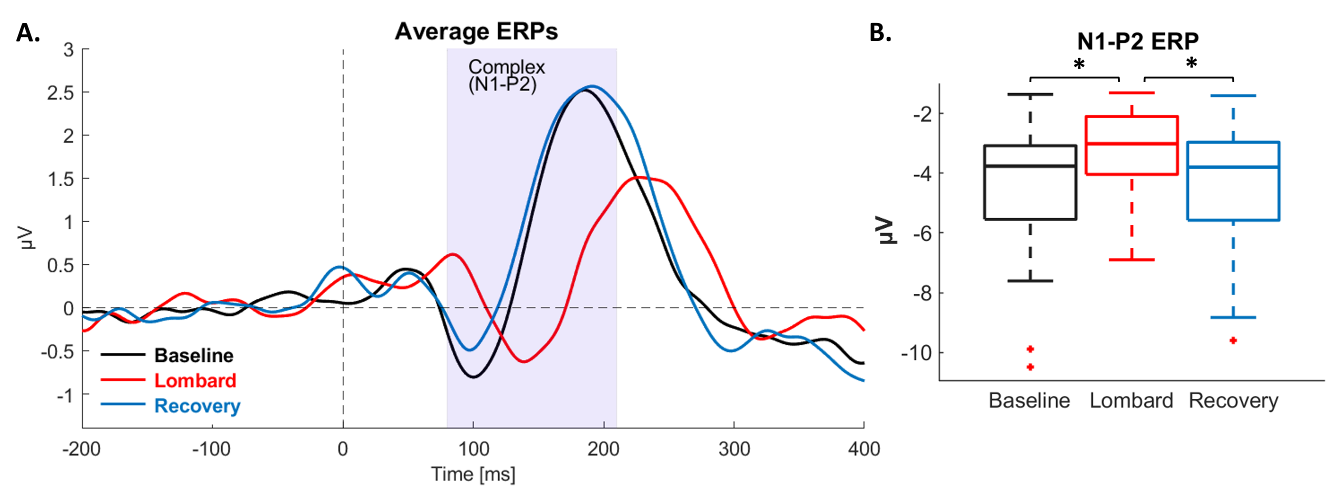

Figure S1. (A) Grand average ERPs across all participants, computed from a cluster of posterior and parietal electrodes (O1, O2, Oz, P1, P2, P3, P4, P5, P6, P7, P8, P9, P10, PO3, PO4, PO7, PO8, POz, and Pz). (B) Mean N1–P2 ERP amplitude for each condition. Asterisks (*) indicate significant differences between conditions (p < 0.05).

Supplementary Analysis of the Main Experiment

Trial-related changes in SPL measures

To examine whether participants showed behavioral adaptation across successive trials in the active task, we fitted a linear mixed-effects model with SPL values for the Lombard condition as the dependent variable and Trial as a fixed effect, including a random intercept for each participant. Assumptions of linearity, homoscedasticity, and normality of residuals were evaluated using residual and Q–Q plots and were reasonably met. Linear mixed-effects models were fitted in R using the lme4 and lmerTest packages.

$$\mathrm{SPL}_{ij} = \beta_0 + \beta_1\,\mathrm{Trial}_{ij} + u_{0j} + \varepsilon_{ij} \tag{1}$$

In the preceding equation, SPL varies as a function of trial number, with β_0 representing the fixed intercept and β_1 representing the fixed effect of Trial, u_{0j} capturing the random intercept for each participant, and ε_{ij} indicating the residual error.

Linear mixed-effects models showed a significant positive effect of Trial in the Lombard condition, indicating a gradual increase in SPL over successive trials. Although the change per trial was small, it was consistent across participants. Random intercepts accounted for between-participant variability.

Condition	Effect	Estimate	SE	df	t	p
Lombard	Intercept	83.10	1.18	20.14	70.26	$< 2 \times 10^{-16}$
	Trial	0.0135	0.0018	1616	7.69	2.49×10^{-14}

Table 1. Linear mixed-effects model fitted for the Lombard condition, predicting SPL values as a function of Trial with random intercepts for participant.

The gradual changes in SPL across trials can be interpreted as reflecting progressive sensorimotor adaptation and feedforward updating during sustained background noise. These changes reflect adaptation processes that develop over time[1]. In the main manuscript, we report effects based on averaged acoustic values, which is more compatible with an ERP-based design that requires averaging a large number of trials per condition. The trial-level analyses are therefore presented as exploratory, to examine how SPL changes within the Lombard condition.

Relation between acoustic measures and N1–P2 amplitude responses

To further examine potential associations between mean acoustic measures and neural responses, we fitted a linear model relating behavioral acoustic measures (SPL, H1–H2, and CPP) to N1–P2 ERP amplitude. Model assumptions were evaluated using residual and Q–Q plots, which indicated acceptable linearity, homoscedasticity, and normality of residuals. In addition, multicollinearity among predictors was assessed using variance inflation factors (VIF), which showed low collinearity between the acoustic measures. The linear models were fitted in R.

$$\mathrm{N1\text{–}P2}_j = \beta_0 + \beta_1\,\mathrm{SPL}_j + \beta_2\,\mathrm{H1\text{–}H2}_j + \beta_3\,\mathrm{CPP}_j + \varepsilon_j \tag{2}$$

In this model, N1–P2 amplitude is regressed on individual acoustic measures, with β_0 indicating the intercept and β_1, β_2, and β_3 representing the effects of SPL, H1–H2, and CPP, respectively. The term ε_j represents the unexplained variance at the participant level.

Predictor	Estimate	SE	t	p
Intercept	-0.69	7.23	-0.10	0.93
SPL	0.07	0.09	0.76	0.46
H1–H2	-0.22	0.29	-0.74	0.47
CPP	-0.27	0.14	-1.88	0.08

$R^2 = 0.17$, Adjusted $R^2 = 0.03$, F-statistic = 1.189, Residual SE = 2.03, df = 17

Table 2. Linear model relating acoustic measures to N1–P2 amplitude in the Lombard condition.

The overall linear model was not statistically significant ($R^2 = 0.17$, $F(3,17) = 1.19$, $p = 0.34$). Together, SPL, H1–H2, and CPP explained approximately 17% of the variance in N1–P2 amplitude. None of the individual predictors reached statistical significance. This result may reflect the limited sample size, which reduces sensitivity to small associations between acoustic and neural measures.

ICA-based decomposition of averaged data

As described in the Methods, the preprocessing pipeline included a second ICA decomposition applied to the averaged ERP data to better separate neural signal from residual noise. Figure S2 shows the ERPs obtained prior to this second ICA step. The overall pattern of the N1–P2 complex are similar to those obtained after applying the second ICA decomposition. The Lombard condition elicits larger amplitudes relative to baseline and recovery, with baseline and recovery showing comparable responses. The second ICA step therefore did not alter the condition differences, but improved waveform morphology while preserving the main findings.

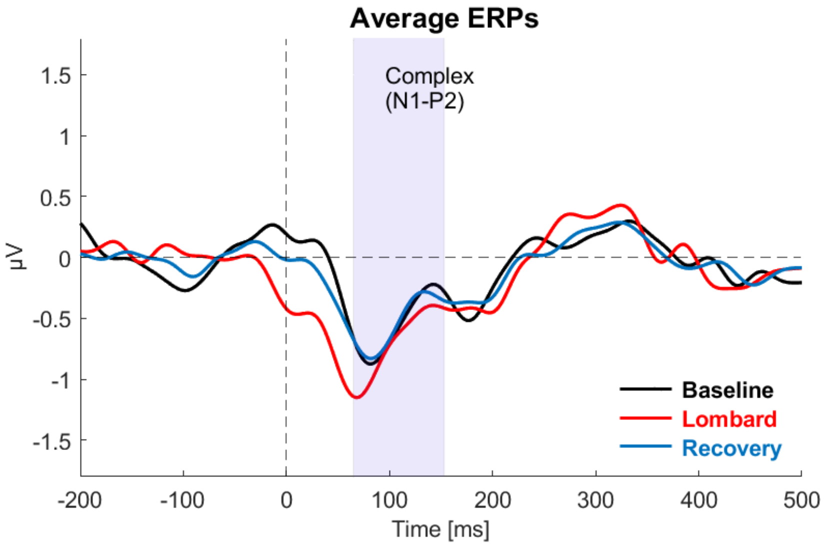

Figure S2. ERP waveforms obtained before applying the second ICA decomposition to the averaged data. All other preprocessing steps and the participant sample were identical to the main analysis.

References

1. Castro, C. *et al.* Modeling voice production and self-perception in noise: Understanding the lombard effect in non-phonotraumatic vocal hyperfunction. *The J. Acoust. Soc. Am.* **156**, 3772–3779, DOI: https://doi.org/10.1121/10.0034544 (2024).
